# Musculoskeletal disorders, associated factors, and literature review for intervention according to gender

**DOI:** 10.47626/1679-4435-2023-1117

**Published:** 2024-09-24

**Authors:** Wilder Alfonso Hernández-Duarte

**Affiliations:** Corporación Universitaria Minuto de Dios (UNIMINUTO), Bogotá, Colombia

**Keywords:** gender identity, musculoskeletal pain, disease prevention, health personnel, review literature as topic, género, dolor musculoesquelético, prevención de enfermedades, personal de salud, literatura de revisión como asunto

## Abstract

**Introduction:**

The relationship of risk factors with musculoskeletal disorders and their intervention is a topic of interest, given their prevalence among workers. Thus, analyzing risk factors from approaches such as the gender perspective may be an alternative.

**Objectives:**

To analyze risk factors in a health entity with a high prevalence of musculoskeletal disorders in upper limbs and to describe possible intervention measures according to scientific evidence, from a gender perspective.

**Methods:**

This is an analytical study. A questionnaire was applied to 93 workers on demographic aspects, presence of factors related to the environment, the task, and the organization, extra-work activities related to musculoskeletal disorders in the upper limbs. Chi-square was used to identify significant relationships between the sex variable and individual, occupational, and non-occupational factors, corroborated by Fisher’s test and prevalence ratio. According to the associations identified, a literature review was carried out to establish possible strategies.

**Results:**

Significant relationships were found between the sex variable and task-related factors such as the presence of repetitive or sudden movements (p < 0.05), supporting postulates of labor segregation. According to the literature consulted, the effectiveness of activities such as physical preparation and adaptation of the workplace under professional guidance, training activities, and breaks is discussed. It is important to review organizational factors.

**Conclusions:**

By identifying significant relationships between the sex variable and task-related factors, the present study contributes to the postulate of labor segregation, in terms of concentration of female labor in activities with particular working conditions. Regarding literature and actions, it is important to generate more studies from this perspective.

## Abstract (pt)

**Introducción:**

La relación de factores de riesgo con desordenes musculoesqueléticos y su intervención es tema de interés, dada su prevalencia entre trabajadores. Así, analizar los factores de riesgo desde enfoques como la perspectiva de género puede ser una alternativa.

**Objetivos:**

Analizar factores de riesgo en una entidad sanitaria con alta prevalencia de desordenes musculoesqueléticos en miembros superiores y describir posibles medidas de intervención según evidencia científica, desde la perspectiva de género.

**Métodos:**

Éste es un estudio de tipo analítico. Se aplicó un cuestionario a 93 trabajadores sobre aspectos demográficos, presencia de factores relacionados con ambiente, tarea y organización, y actividades extralaborales vinculadas con desórdenes musculoesqueléticos en miembros superiores. Mediante chi cuadrado, se realizó la identificación de relaciones significativas entre la variable sexo y factores individuales, intralaborales y extralaborales, corroboradas por prueba de Fisher y razón de prevalencias. Según las asociaciones identificadas, se efectuó revisión de literatura para establecer posibles estrategias.

**Resultados:**

Se encontraron relaciones significativas entre la variable sexo y factores de la tarea como la presencia de movimientos repetitivos o súbitos (p < 0,05), apoyando postulados de segregación laboral. Según la bibliografia consultada, se discute la eficacia de actividades como preparación física y adecuación del puesto de trabajo bajo orientación profesional, actividades de capacitación y pausas. Es importante revisar factores organizacionales.

**Conclusiones:**

Al identificarse relaciones significativas entre la variable sexo y factores de la tarea, se aporta al postulado de segregación laboral en cuanto a concentración de mano de obra femenina en actividades con condiciones de trabajo particulares. Sobre la literatura y las acciones, es importante generar más estudios desde esta perspectiva.

## INTRODUCTION

Musculoskeletal disorders (MSD) consist of a group of diseases characterized by an abnormal condition of muscles, tendons, nerves, joints, or ligaments that results in changes in motor or sensory function.^1^ This obviously have an impact on quality of life, producing temporary or permanent disabilities, inability to perform, increased costs for workers’ compensation, reduced productivity and rentabilidad.^2^

According to the results of the Third National Survey on the Occupational Safety and Health Conditions in the General System of Occupational Risks, in Colombia the leading risk factors are those related to activities that involve frequent hand or arm movements, maintaining the same posture for all or the majority of working hours, and prolonged postures that could cause tiredness or pain, all of them related to the physical demands of the activity.^3^

However, the literature has also described other risk factors that have shown to be somewhat associated with the onset of these changes, such as the greater susceptibility of the female population. For instance, it has been described that the impact of housework activities increases the possibility of suffering from MSD, especially in areas such as neck and upper limbs.^4,5,6^

The aforementioned reveals the need of analyzing working conditions and additional factors from the gender perspective, understood as those scenarios that make “reference to the social differences between men and women that have been learned, change over time, and present great variations both between different cultures and within the same culture”^7^ and, under this principle, male and female workforces tend to concentrate in activities with certain characteristics. Studies on MSD conducted under this point of view found differences in the affected area, as well as different risk factors depending on the work activities performed by men and women.^8^

Based on the foregoing, an analysis of working conditions from the gender perspective is proposed in a health institution that had a high prevalence of MSD in upper limbs and a consequently high impact on absenteeism, with reports of 373 disabilities from these diseases in 2019. Furthermore, possible intervention measures are described according to findings from the scientific literature.

The present study aimed to conduct an analysis on working conditions considering other points of view, such as that of the gender perspective, in the context of occupational safety and health management, and guiding proper intervention based on previous findings.

## METHODS

The present study was conducted using an analytical cross-sectional approach, since it aimed to examine risk factors associated with MSD in upper limbs, from the gender perspective, and subsequently to identify intervention actions based on scientific evidence.

### POPULATION AND INCLUSION CRITERIA

This study was conducted through a non-probabilistic convenience availability sampling. The participant population consisted of health care employees of both sexes registered under the special regime at a health institution located in the city of Bogotá, Colombia. In total, 93 workers out of 121 participated, including nursing, physical therapy, medical, bacteriological, and dental professionals. The study included individuals who accepted to participate by answering the survey.

### INSTRUMENTS

The instrument for information collection was a self-administered questionnaire applied through an online form, after content validation, in order to investigate sociodemographic aspects and presence of risk factors related to the task the work environment, and the organization; as well as non-occupational aspects that, according to the scientific literature, have related to MSD in upper limbs. The instrument was applied under supervision of participants in the research seedbed entitled “Management of working and health conditions,” by the Minute of God University Corporation (*Corporation Universitária Minuto de Dios,* UNIMINUTO).

### PROCEDURE

The research proposal was presented to the health institution, and, after its due approval, the participating population was informed about the aim of the study. Subsequently, they were given the informed consent form, which ensured the privacy of the collected information. The questionnaire was applied to the working population of the indicated positions through a form, with the respective supervision of the participants in the research seedbed.

Once information was consolidated in a database, it was reviewed and filtered. Subsequently, data were analyzed and duly processed by software, in order to determine significant statistical relationships.

Based on the identified findings, a search was conducted for studies about actions and strategies on databases such as Science Direct, Virtual Health Library (VHL) Regional Portal, Cochrane Library, MEDLINE, National Library of Medicine (NIH), and SCOPUS.

The search strategy used the following Medical Subject Headings (MeSH) terms, searched on Descritores em Ciências da Saúde (DeCS): gender, primary prevention, musculoskeletal pain, healthcare workers, repetitive movements, upper limbs. Subsequently, search equations were established, such as “primary prevention and gender and musculoskeletal pain and upper limbs,” both in English and Spanish languages, which were applied on the previously described databases.

The articles that met inclusion criteria and the theme of interest were analyzed to find possible intervention actions.

### ANALYSIS OF INFORMATION

The collected information was processed on the SPSS software (licensed by UNIMINUTO) to analyze frequency distribution and to apply non-parametric statistical tests such as the chi-square test, in order to evaluate the relationship between the different variables of occupational and non-occupational factors and the variable sex, all of which were quantitative variables. The Fisher’s exact test was applied to corroborate the statistical relationships, and prevalence ratios (PR) were calculated to identify possible associations, considering significant relationships those with a confidence interval equal to or greater than 95% (IC ≥ 95%).

In order to determine possible actions based on the statistical relationships identified, the results obtained from the literature review were consolidated in an Excel database to determine which ones would be the intervention measures targeting the worker, the environment, and the source of risk.

### ETHICAL CONSIDERATIONS

Finally, it bears highlighting as ethical considerations the facts that approval was obtained from the health institution and that the surveyed population was informed about the aim of the study through the informed consent form, ensuring information confidentiality.

## RESULTS

The surveyed population totaled 93 employees of a health institution. As shown in Table 1, most participants were women, which accounted for almost 84% of the sample. A third of the population consisted of nurses, and 57% had a romantic partner.

**Table 1:** Demographic characteristics of the working population of a health institution

Variables	n	%
Sex		
Female	78	83.9
Male	15	16.1
Current position		
Nurse	31	33.3
Physical therapist	17	18.3
Physician	17	18.3
Bacteriologist	15	16.1
Dentist	13	14.0
Marital status		
Married	53	570
Single	32	34.4
Separated	7	7.5
Widowed	1	1.1

With regard to working conditions, Table 2 shows that 54.8% of workers reported performing identical or similar elbow movements every few seconds, and 50.5% of them reported performing strong sudden writs movements along with uncomfortable postures. This study also found that 41.94% of the surveyed population performed tasks that involved handling objects and tools weighting 2 kg or more.

**Table 2:** Characteristics of working conditions reported by the working population of the health institution

Variables		n	%
Task-related factors			
During working hours, do you perform Identical or similar elbow movements every few seconds?	No	42	45.2
	Yes	51	54.8
Do your tasks often involve strong or sudden rapid writs movements combined with awkward posture?	No	46	49.5
	Yes	47	50.5
During your tasks, do you often handle objects or tools weighting 2 kg or more?	No	54	58.1
	Yes	39	41.9
Environmental factors			
Are there extreme temperature variations during your working hours?	No	47	50.5
	Yes	46	49.5
Is the level of lighting low in your workplace?	No	81	87.1
	Yes	12	12.9
Factors related to the organization			
Does workload require you to work overtime?	No	18	19.4
	Yes	75	80.6
Does pace of work allow you to take breaks?	No	59	63.4
	Yes	34	36.6

In relation to conditions associated with the work environment, it was observed that extreme temperature variations during working hours were reported by 49.5% of the population.

Finally, as for organizational factors, it is worth highlighting that 80.6% of the sample reported working overtime because of high workload.

According to Table 3, relative to other associated factors, it was found that 39.8% of the sample exercise once or twice a week, while 35.5% of respondents do not practice any physical activity.

**Table 3:** Characterization of non-occupational risk factors reported by the working population of a health institution

Variables	Times a week	n	%
Weekly frequency of exercise or physical activity for more than 30 minutes	1-2	37	39.8
	3-4	13	13.9
	5 or more	10	10.8
	Do not practice	33	35.5
Weekly frequency of cell phone use for more than 2 hours daily	1-2	19	20.4
	3-4	25	26.9
	5 or more	42	45.2
	Do not use it	7	7.5
Weekly frequency of computed use outside work	1-2	45	48.4
	3-4	8	8.6
	5 or more	12	12.9
	Do not use it	28	30.1

In relation to cell phone use for more than two hours daily, 45.2 % of workers who used it more than five times a week for this time interval. Almost half of the population uses the computer outside working hours only once to three times a week.

Demographic variables, working conditions, and non-occupational variables were associated with the variable position. Statistically significant relationships were observed with current job (p < 0.01) (Table 4).

**Table 4:** Relationship between the variable sex and exposure to risk factors

Variables	Chi square	p-value*
Demographic variables		
Sex/current position	16.47	0.002
Working conditions		
Task-related aspects		
Sex/During working hours, do you perform identical or similar elbow movements every few seconds?	5.73	0.017
Sex/Do your tasks often involve strong or sudden rapid writs movements combined with awkward posture?	4.07	0.043
Sex/During your tasks, do you often handle objects or tools weighting 2 kg or more?	3.53	0.060
Environmental aspects		
Sex/Are there extreme temperature variations during your working hours?	3.71	0.053
Organizational aspects		
Sex/Does workload require you to work overtime?	1.84	0.174
Sex/Does work rhythm allow you to take breaks?	2.17	0.141
Non-occupational factors		
Sex/How many times do you perform physical activity per week?	2.74	0.431
Sex/ How many times do you use the cell phone more than 2 hours daily per week?	4.34	0.237

* ≥ 95% confidence interval.

Among intra-occupational conditions, significant relationships were found only with aspects of the task such as presence of high-intensity movements (p < 0.05) and sudden movements along with awkward posture (p < 0.05). There were no significant relationships with the most frequent non-occupational factors described in this study.

However, in order to corroborate significant relationships, the Fisher’s exact test was applied, and PR was calculated to detect possible associations between the indicated variables. Statistically significant relationships were found between the variable sex and exposure to identical or similar elbow movements (Fisher: p < 0.05), as well as between worker’s sex and exposure to this risk factor (PR = 4.2; 95%CI 1.2-14.3). When assessing PR according to sex, exposure to this risk factor was slightly more probable for women than for men (PR for women = 1.2; 95%CI: 1.03-1.52).

Conversely, there was significant relationship between the variable sex and exposure to sudden wrist movements combined with awkward posture, yielding a value slightly outside the 95%CI in the Fisher’s exact test, with similar results when association strength was determined using PR (PR = 3.4; 95%CI: 0.9-11.5). However, there was a slight change when the probability of exposure to this risk factor was assessed by sex (PR for women = 1.2; 95%CI: 1.0-1.4) (Table 5).

**Table 5:** Corroboration of relationships between the variable sex and exposure to risk factors and its association

Variables	Fisher’s exact test*	PR	95%CI
Demographic variables			
Sex/current position	NA	Nurse: 1.2	0.2-7.7
		Physical therapist: 2.9	0.2-36.1
		Bacteriologist: 5.5	5.5-5.5
		Physician: 0.2	0.0-1.2
		Dentist: 0.8	0.1-5.1
Working conditions			
Task-related aspects			
Sex/ During working hours, do you perform identical or similar elbow movements every few seconds?	0.023	4.2	1.2-14.3
		Women: 1.2	1.0-1.5
		Men: 0.3	0.1-0.8
Sex/Do your tasks often involve strong or sudden rapid writs movements combined with awkward posture?	0.052	3.4	0.9-11.5
		Women: 1.2	1.0-1.4
		Men: 0.3	0.1-1.0
Sex/During your tasks, do you often handle objects or tools weighting 2 kg or more?	0.086	3.4	0.9-13.1
		Women: 1.2	1.0-1.4
		Men: 0.3	0.1-1.1
Environmental aspects			
Sex/ Are there extreme temperature variations during your working hours?	0.089	3.2	0.9-10.9
		Women: 1.2	1.0-1.4
		Men: 0.4	0.1-1.1
Organizational aspects			
Sex/Does workload require you to work overtime?	0.280	0.2	0.0-2.1
		Women: 0.8	0.7-1.0
		Men: 3.4	0.5-24.0
Sex/Does work pace allow you to take breaks?	0.150	0.4	0.1-1.4
		Women: 0.9	0.7-1.1
		Men: 1.9	0.8-5.0
Non-occupational factors			
Sex/How many times do you perform physical activity per week?	NA	Do not perform: 1.8	0.3-11.7
		Once-twice: 1.6	0.3-9.8
		3-4 times: 0.6	0.0-4.0
		5 times or more: 1.7	0.2-12.4
Sex/How many times do you use the cell phone more than 2 hours daily?	NA	Do not use: 0.2	0.0-1.2
		Once-twice: 1.4	0.3-7.7
		3-4 times: 0.9	0.2-3.4
		5 times or more: 1.1	0.3-4.5

** Prevalence ratio.

NA = not applicable.

* ≥ 95% confidence interval.

Table 6 presents the description of studies applying the search equations previously defined in the databases and considering the findings that had previously shown to have significant relationships. This description included possible actions targeting the worker and the source of risk, but no sufficient evidence was found to guide measures targeting the work environment.

**Table 6:** Intervention measures to consider, according to the identified findings

Author	Title	Database	Strategy
Hayes et al.^16^	An international review of musculoskeletal disorders in the dental hygiene profession	Science Direct	The authors showed that a detailed analysis of the task would potentially help prevent musculoskeletal pains by identifying what joints are more often used in non-neutral positions, which allows for proposition of specific educational and prevention strategies. The proper use of lights, mirrors, and finger supports during instrumentation reduces the risk for development of musculoskeletal pain, in addition to preventing muscle fatigue.
Soler-Font et al.^20^	Multifaceted intervention for the prevention and management of musculoskeletal pain in nursing staff: Results of a cluster randomized controlled trial	NIH	The authors performed an intervention program directed to the nursing staff comprising three evidence-based components (participatory ergonomics, health promotion activities, and case management), including two groups named intervention and control, both of which received usual occupational health care. Musculoskeletal pain and work functioning data were collected at baseline and at 6- and 12-month follow-up. Results showed a statistically significant decrease of the risk for neck, shoulder, and upper back pain in the intervention group, compared to the control group. A reduction of low back pain was also observed, though non statistically significant.
Hoe et al.^19^	Intervenciones ergonómicas para la prevención de los trastornos musculoesqueléticos de miembros superiores y cuello relacionados con el trabajo en oficinistas	Cochrane	First of all, the authors found that use of arm support or computer mouse based on neutral posture, may or may not prevent work-related musculoskeletal pain of the neck and the shoulder. With regard to workstation adjustment and sit-stand desks, they do not have an effect on upper limb pain compared to no intervention.
Nye et al.^14^	Prevention and reduction of musculoskeletal pain through char-side stretching among dental hygiene students	MEDLINE	A series of upper-limb chair-side stretching exercises were completed prior to beginning each clinical session, for approximately 10 weeks, in order to reduce and prevent musculoskeletal pain. It was observed that most participants felt that chair-side stretching exercises neither improved nor worsened their musculoskeletal pain. However, more than one half of the participants felt that the exercises helped increase their conscious level regarding ergonomic practices while delivering patient care. Similarly, findings from the study suggest that side-chair stretching exercises performed consistently on weekly basis may be beneficial in reducing and preventing musculoskeletal pain, particularly within the hand and wrist region.
Kennedy et al.^15^	Systematic review of the role of occupational health and safety interventions in the prevention of upper extremity musculoskeletal symptoms; signs; disorders; injuries; claims and lost time	NIH	The authors of this systematic review found four studies that evaluated exercise programs. The exercise interventions were similar; they involved initial training on exercises (by a physical therapist), followed by an independent exercise program done either during working hours or at home. The four exercise programs included a variety of activities including strengthening, stretching, coordination, relaxation, and stabilization exercises. Overall, these studies provide mixed evidence that exercise programs have an effect on upper extremity musculoskeletal pain outcomes. Ergonomics program combined with exercise. The authors of the systematic review identified three studies that evaluated the combination of these programs. Of these studies, one found significant results for this investigation, showing positive outcomes for neck, shoulders, and elbows, and no effect for wrists/hands. The aim of this study was to compare the effects of two different intervention models for work with visual display units (E = redesign measures for the environment only, ET = redesign measures for both the environment and work techniques) on neck, shoulder, and arm symptoms. Work posture, monitor viewing, muscular activity, and musculoskeletal pain were measured before and after a 7-month intervention. Difference was statistically significant between the groups for the change in shoulder flexion (p = 0.0134) and the muscular activity of right trapezius (p = 0.04109) and right extensor carpi radialis (p = 0.0379) in the pre- and postintervention measurements. The reduction of pain symptoms in the neck (p = 0.0073), shoulders (p = 0.0071), and elbows (p = 0.0490) was greater in the ET group than in the E group. The effect of an array of workstation adjustments were analyzed in four studies, in which individual workstation adjustments were performed by a therapist or technician with the goal of reducing postural stresses. All studies found no effect of workstation adjustments on upper extremity musculoskeletal pain outcomes. Furthermore, these studies provide strong evidence that workstation adjustments alone have no effect on upper extremity musculoskeletal pain outcomes. The systematic review also identified a high-quality ergonomic program combined with workstation adjustments that found a positive effect on the elbow/forearm and no effect on the neck, shoulder, and wrist/hand. Finally, this review assessed three studies that observed positive effects for right upper extremity self-report outcomes. Given the right-handed dominance, the team considers these health effects as important. Moreover, the review identified four studies that implemented different types of training programs ranging from a single session to multiple participatory training sessions. The training duration varied from a 10-minute personal follow-up after receiving an information pamphlet to a 1-hour ergonomics lecture. Together, these studies provide mixed evidence that ergonomics training has an effect on upper extremity musculoskeletal pain outcomes. One high-quality study found a positive effect on the elbow/forearm and no effect on the neck, shoulder, and wrist/hand. This single high-quality study provides limited evidence that ergonomics training plus workstation adjustments have a positive effect on upper extremity MSC outcomes. Three studies evaluated arm supports: two high quality studies found positive and no effect, and one medium quality study found no effect. Positive effects were found in both high quality studies for right upper extremity self-report outcomes. Given the right-handed dominance, the team considers these health effects as important. These studies provide moderate evidence that arm supports have a positive effect on upper extremity MSC outcomes. Rest breaks and supplementary breaks. The systematic review described four studies that evaluated the effects of rest breaks: one found to effect with a 5-minute break every 35 minutes, and the other three found positive or no effect, depending on the rest break pattern. For the positive findings, the break patterns were either a 5-minute break every hour or a 30-second break every 20 minutes.
Holzgreve et al.^17^	Office work and stretch training (OST) study: effects on the prevalence of musculoskeletal diseases and gender differences: a non-randomised control study	SCOPUS	Application of a non-randomized study of a stretching program to 252 employees (110 women and 142 men) of a German car manufacturer completed 22-24 training sessions 3 months. The gender analysis revealed that women were, in general, more often affected by musculoskeletal complaints than men in the neck and feet. Both sexes had significant reductions of musculoskeletal pain in the most commonly affected regions.
Ziam et al.^22^	Musculoskeletal disorder (MSD) prevention practices by nurses working in health care settings: Facilitators and barriers to implementation	Science Direct	The authors report on individual and organizational factors that may influence nurses’ implementation of MSD prevention practices. A total of 339 questionnaires were completed, and the results revealed that nurses have the required knowledge on preventive practices, but have difficulty applying them in their professional context, due to organizational factors such as management support, organizational culture, feedback mechanisms, and training that is adapted to the work environment.
Albanesi et al.^18^	Interventions to prevent and reduce work-related musculoskeletal injuries and pain among healthcare professionals. A comprehensive systematic review of the literature	Science Direct	The authors provided a comprehensive description of interventions for preventing and reducing MSD among healthcare professionals through a systematic review. It was observed that individual interventions such as physical exercise were effective in reducing pain, that task-specific and work organization interventions could prevent certain injuries, and that significant reduction of both injuries and pain resulted from multifactorial interventions.
Rodríguez-Blanes et al.^21^	The influence of information on the prevention of occupational risks and ergonomic requirements in the development of non-traumatic osteomuscular diseases of the shoulder – a pilot study	Science Direct	The authors analyzed the association between MSD of the shoulder and workers’ knowledge of the risks at the workplace and preventive measures developed there, as well as the association with ergonomic requirements. Lower probability of shoulder injury was associated with male sex, working hours > 9 hours, as well as having information on occupational risk at the workplace, the knowledge of the prevention plan, period medical examinations.

MSC = musculoskeletal conditions; MSD = musculoskeletal diseases; NIH = National Library of Medicine.

In relation to measures targeting the worker, the present search found studies that evaluated the impact of warm-up and physical conditioning exercises. Regarding the source, previous investigations evaluated workplace adjustment, which involved furniture and due instruction on its proper use. Moreover, there were mentions on the relevance of assessing organizational factors for the implementation of interventions. Further details on the matter can be found in the discussion section.

## DISCUSSION

Although the present study observed most of the participating population was female and there was a significant relationship between the variable sex and current job in the participating population, no association was found. However, coherent with previous studies on demographic characterization, it is worth noting that the highest percentage of healthcare workers was female. The fact that the positions under study are performed mainly by women is in agreement with findings reported by the Colombia’s Ministry of Health and Social Protection,^9^ which showed that, for 2021, 79% of professionals registered in the National Single Registry of Human Talent in Health *Registro Único Nacional de Talento Humano en Salud,* ReTHUS) are women and work in fields such as nutrition and dietetics, occupational and respiratory therapies, speech therapy, physical therapy, bacteriology, and nursing, accounting for approximately 90% of nurses. Medicine is the only health profession in which the proportion of women is lower compared to men (47 versus 52%).

In view of the foregoing, there has been evidence of a relationship between presence of MSD and female population in the health care sector. Using a logit model to determine the presence of musculoskeletal disorders in worker in the hospital sector, Bedoya-Marrugo et al.^10^ found that sex and position were factors that had a significant effect on musculoskeletal conditions among the workers participating in the study (p < 0.01). In terms of the relationship between the variable sex and musculoskeletal disorders, the same authors found that 80% of women had MSD, and only 55% of men did not present with musculoskeletal conditions. This could be explained by the specific working conditions related to the tasks performed by the participants in their positions, an aspect that will be explained next.

It is necessary to bear in mind that this study found significant relationships only between the variable sex and task-related factors such as presence of high-intensity movements, combined with awkward upper limb postures, which is corroborated by the exact Fisher’s test and PR for some of these factors. In light of the foregoing, previous studies suggest that the high frequency of MSD among female workers may be related to cultural aspects that promote the concentration of the female labor in activities with certain working conditions may facilitate the onset of these diseases, a situation known as labor segregation. In other words, labor is concentrated in work activities that particularly require using upper limbs in highly frequent movements or high levels of hand strength.^5,6,11,12,13^

Based on the statistically significant relationships found, a search was conducted for studies that provided guidance on intervention actions, most of which consisted of activities targeted at the worker and source.

With regard to the worker, the literature presented studies on practices that involved upper limb warm-up, prior to beginning work activities, associated with training activities.

Nye et al.^14^ described a strategy consisting of a series of upper-limb chair-side stretching exercises completed prior to beginning of workers’ activities. These authors observed that most participants felt that chair-side stretching exercises neither improved nor worsened their musculoskeletal pain. However, more than one half of the participants felt that the exercises helped increase their conscious level regarding ergonomic practices.

In their systematic review, Kennedy et al.^15^ found studies on the effects of exercise programs on upper limb pain, following an activity routine, either under professional supervision or independently at work and at home. This review also found studies that evaluated the combination of ergonomics intervention programs with exercises, with results showing a greater impact on those groups that performed the redesign of workstations plus training on work techniques.

The literature review by Hayes et al.,^16^ in turn, observed that a detailed analysis of the task would potentially help prevent musculoskeletal pain by identifying what joint are most often used in non-neutral positions, which allows for planning of specific educational and prevention strategies.

Holzgreve et al.^17^ discussed the execution of physical training programs and their impact on a working population, in the context of health and healthy lifestyle promotion. For this purpose, they conducted a non-randomized study of stretching program performed by 252 employees (110 women and 142 men) of a German car at a frequency of 22-24 training units for 3 months. Data analysis by sex found that women were, in general, more often affected by musculoskeletal complaints than man in the neck and feet. Both sexes had significant reduction of complaints after intervention. A literature review by Albanesi et al.^18^ also described reports related to the application of physical exercise, but also mentioned cognitive-behavioral therapy and neuromuscular exercises.

With regard to measures targeting the source, a systematic review conducted by Hoe et al.^19^ stated that there is inconsistent evidence on the adjustment of elements in the work environment to prevent the onset of MSD.

In their systematic review, Kennedy et al.^15^ described the findings of studies in which the intervention measure was workstation adjustments supervised by a professional specialized in the area. These studies found no effects of workstation adjustments on upper extremity musculoskeletal pain outcomes. Furthermore, these studies provided strong evidence that workstation adjustments alone have no effect on upper extremity musculoskeletal pain outcomes.

In their literature review, Albanesi et al.^18^ described studies on the application of mechanical aids plus theoretical training combined with exercises and did not report any change in the rate of MSD symptoms.

However, the study by Hayes et al.^16^ observed that the proper placement of the elements of the workplace for the execution of tasks among dental professionals reduces the risk for musculoskeletal pain and prevents muscular fatigue.

Soler-Font et al.^20^ conducted an intervention program directed to the nursing staff that comprised three evidence-based components: 1. Participatory ergonomics, 2. Health promotion activities, and 3. Case management, which was performed through a case-control study. Musculoskeletal pain and work functioning data were collected at baseline and at 6- and 12-month follow-up. The intervention group showed a statistically significant decrease of the risk in neck, shoulders and upper back pain, compared to the control group. A reduction of low back pain was also observed, though not statistically significant.

The participatory ergonomics program was developed in three phases: the first of which consisted of identifying musculoskeletal symptoms, occupational risk factors, and level of musculoskeletal risk. The second phase, that of treatment, involved a joint committee that held weekly meetings to propose preventive measures. Finally, the last phase consisted of the execution of preventive measures that included organizational, structural, technical, training/information improvements in the workplace.

The healthy lifestyle promotion program was developed to encourage healthy lifestyles among health workers, including activities such as Nordic walking, mindfulness, and healthy diet based on the Mediterranean diet disseminated on a website.

The tailored case management program, in turn, aimed for early detection of musculoskeletal conditions and support of return to work.

In their systematic review, Kennedy et al.^15^ found four studies that implemented different types of training programs ranging from a single session to multiple participatory training sessions. The training duration varied from a 10-minute personal follow-up after receiving an information pamphlet to a 1-hour ergonomics lecture. Together, these studies provide mixed evidence that ergonomics training has an effect on upper extremity musculoskeletal pain outcomes.

Rodríguez-Blanes et al.^21^ analyzed the association between the existence of musculoskeletal disorders of the shoulder and the workers’ knowledge of the risks at the workplace and preventive measures developed there, as well as the association with ergonomic requirements. These authors found a lower probability of presenting injuries of this joint among men and among those who had information on the risks associated with the workplace, the knowledge on the prevention plan, and performed periodic medical examinations, among others. However, the authors observed that, in their literature review, there were limited evidence on the effectiveness of informative or educational programs.

In relation to rest breaks, Kennedy et al.^15^ also found investigations on the subject. Four studies assessed the effects of rest breaks, of which one found no effect with a 5-minute break every 35 minutes, whereas the others found positive or no effect, depending on the rest break pattern. For the positive findings, the break patterns were either a 5-minute break every hour or a 30-second break every 20 minutes. In their respective studies, Hoe et al.^19^ and Hayes et al.^16^ also observed that supplementary breaks may reduce upper limb discomfort or muscular fatigue.

Ziam et al.^22^ described in their study individual and organizational factors that may influence the implementation of MSD preventive practices among nursing professionals in the Quebec region. It was shown that this population has the required knowledge on MSD prevention practices, but has difficulty applying them in their occupational context, due to organizational factors such as management support, organizational culture, feedback mechanisms, and training that is adapted to the work environment. Good channels of communication and a good organization culture may have a significant role in preventing MSD and increasing workers’ capacity.^18^

## CONCLUSIONS AND RECOMMENDATIONS

A gender analysis of risk factors for upper limb MSD at a health entity with a high prevalence of these diseases among healthcare professions showed statistically significant relationships between the variable sex and task-related factors such as high-intensity movements (p < 0.05) and combination of upper limb sudden movements with awkward postures (p < 0.05), whose significance was corroborated for exposure to high-intensity movements, as well as their association. These findings contribute to the postulate of labor segregation, in terms of concentration of female or male labor in activities that demand the use of certain body segments and the presence of high-intensity physical demands, such high-intensity movement, forced postures, or grips requiring strength.

As indicated by the foregoing, it is recommended to develop future studies that allow corroborating or aggregating more elements to the results obtained by this investigation, e.g., with larger samples sized and in other economic activities.

With regard to possible intervention measures, based on the identified findings, the scientific literature shows the discussion on the lightness of evidence and analyzes the need of performing warm-up and stretching activities for upper limbs and neck, which are measures targeting the worker.

The literature also examined the relevance of reviewing and adjusting the workstation and its elements under the guidance of expert professionals, in addition to the implementation of educational and training activities and rest break, all of them targeting the source. The role of organizational factors in the application of these measures by the working population is also discussed.

Conversely, although possible intervention measures were found in the scientific literature, they were considered according to the associations found; hence, there are still concerns regarding the availability of studies about interventions from the gender perspective.
